# Early clinical management of severe burn patients using telemedicine: a pilot study protocol

**DOI:** 10.1186/s40814-020-00637-7

**Published:** 2020-07-04

**Authors:** Maxim Moreau, Guy Paré

**Affiliations:** HEC Montréal, 3000 Chemin de la Côte-Ste-Catherine, Montréal, Québec H3T 2A7 Canada

**Keywords:** Telemedicine, Burn medicine, Patient transfers, Clinical management, Outcomes

## Abstract

**Background:**

Emergency physicians are responsible for assessing the severity of a patient’s burns, which determines whether the patient needs to be transferred to a burn center. Such a proper assessment represents a daunting task because severe burn injuries are rare. Inaccurate estimates often result in unjustified and costly transfers and unneeded fluid resuscitation and assisted ventilation procedures. Telemedicine offers a solution to these challenges. The present pilot study aims to investigate the feasibility, acceptability, and potential value of a large telemedicine initiative at the University of Montreal Health Center’s burn center and its network of referring hospitals.

**Methods:**

A three-stage study protocol is proposed to achieve this objective. First, a proof of concept phase will assess the technical feasibility of telemedicine at one referring hospital with a high volume of patient transfers. Second, the organizational and human feasibility of the project will be evaluated in four referring medical centers. All teleconsultation sessions will be analyzed using the WHO’s telemedicine implementation model. The third phase will consist of evaluating the potential impacts of telemedicine in a subset of 10 referring hospitals. The quality of communications between referring physicians and specialists will be assessed using semi-structured interviews. A pre-test/post-test with a comparison group design will be used to assess the effects of telemedicine on patient transfers, ventilation procedures, patient complications, mortality, length of ICU stay, and additional surgical procedures. The economic viability of telemedicine will be assessed using a cost-minimization approach.

**Discussion:**

The telemedicine initiative is expected to yield positive and significant outcomes that are relevant to a wide range of medical centers that already use or are considering using a similar technology. The contribution of this pilot study lies in its ability to reveal technological, organizational, and human barriers and provide a preliminary assessment of the clinical and economic value of a large-scale telemedicine initiative in the context of burn medicine.

## Background

Exposure to intense heat or harmful sources of electricity or chemicals can damage the skin and adjacent tissues. Severe burn injuries, which have historically been defined as > 20% of the total body surface area [1], can be devastating. Despite a decrease in their incidence around the world, severe burns represent traumas with high mortality rates [2, 3]. In the absence of appropriate and rapid case management, serious burns may lead to systemic deterioration, organ failure, and even death [2, 4]. Emergency physicians are responsible for making a proper assessment of the severity of a patient’s burns and then determining whether fluid resuscitation and assisted ventilation procedures are necessary. Such a proper assessment is key to determining whether the patient needs to be transferred to a burn center [5, 6].

Although various techniques can be used to assess burn area and depth [7], in practice, visual examination is the technique most often used. Unfortunately, the literature reports significant discrepancies between burn severity estimates by emergency physicians and those made by specialists at burn centers [8–10]. Inaccurate estimates often result in overtriage which, in turn, leads to unjustified transfers of patients with minor burns to burn centers. The rate of unjustified transfer can be relatively high, sometimes reaching one third of all transfers [11]. The literature also reports that an inaccurate assessment of burn severity can lead to the patient receiving unneeded fluid resuscitation [12, 13], at high cost to the health system [9, 14].

In order to address these challenges and streamline the process of patient transfer to the burn center, senior administrators at the University of Montreal Health Center (called CHUM in French) have decided to invest in a large-scale video-based telemedicine project. At this time, mobile phones are the predominant communication technology used by referring physicians and CHUM’s consulting specialists. Whereas the use of personal mobile devices may enrich communication between physicians and improve clinical decision making, it also raises several issues with regard to the security and confidentiality of medical information, the traceability of the discussions between providers, and the transfer and storage of medical notes and images in the electronic health record (EHR) system of the participating hospitals [15, 16].

Our research team’s assistance was first requested to perform an independent, yet comprehensive assessment of existing practices and problems. To this end, we collected and then analyzed three data sources: (1) semi-structured interviews with two members of the burn center’s medical team and six referring physicians who had recently contacted the burn center; (2) data from Quebec’s trauma registry information system (called SIRTQ in French); and (3) the burn center’s internal documents. Our analysis revealed five major problems with current work methods and tools:

- *Non-compliance with the ethics rules of the Quebec College of Physicians*. The College mentions the need to comply with practices set forth in *An Act to establish a legal framework for information technology*, particularly with respect to patient confidentiality and recordkeeping. This framework specifies that (a) images of a patient taken with a smartphone must be duly erased from the device after use; (b) transmission of patient information and data storage must be secure and confidential; and (c) the information sent (e.g., digital photos, video sequences, medical notes) must be uploaded to the patient’s electronic medical record.
- *Imprecise assessment of the body surface area affected*. The analysis of the data in the SIRTQ database found differences of 10 to 45% in the estimates made of body surface area (2016 and 2017).
- *Unjustified transfers of patients to the burn center*. According to the medical specialists interviewed, a stay in the center of less than 24 h indicates an unjustified patient transfer. The rate of unjustified transfers was approximately 11% in 2016 and 12% in 2017.
- *Unjustified use of assisted ventilation procedures*. According to the medical specialists, withdrawal of assisted ventilation less than 24 h after transfer to the center indicates unjustified intubation and mechanically supported ventilation of the patient. The rate of unjustified ventilation from 2012 to 2017 was 39%.
- *Expenses incurred for unjustified procedures*. The actual cost associated with transferring a patient to the burn center plus 5 days of hospitalization is estimated at CA$13,330 (excluding fee-for-service expenses). An additional CA$10,540 must be added in cases of aeromedical evacuation (representing close to 2% of the transfers to the center). Patients transferred under assisted ventilation require assistance on the ambulance (a nurse, respiratory therapist, or physician) in addition to the usual paramedics, resulting in higher costs.

Next, our research team was asked to assess the feasibility, acceptability, and potential value of the telemedicine initiative. We developed a three-stage pilot study which we describe next. Our specific objectives are twofold: (1) to identify the technological, organizational, and human issues that are likely to surface before and during the deployment of telemedicine at the burn center and its referring hospitals; and (2) provide a preliminary, yet comprehensive assessment of the clinical and economic outcomes associated with the use of telemedicine in this context.

## Methods

### Study setting

CHUM is one of the largest medical centers in Canada. It is a public not-for-profit hospital whose primary mission is to provide inpatient and ambulatory care to its immediate urban clientele, as well as specialized and ultra-specialized services to the broader metropolitan and provincial population. The burn center at CHUM is one of the two referral centers for severe burn victims in Quebec, Canada (See Supplementary Table 1, Additional File 1). It admits approximately 150 patients per year, with a higher pace of admissions in the summer. Before a patient can be transferred, a physician at the referring site must contact a specialist at the burn center to obtain prior consent. The referring provider can take advantage of the specialist’s expertise for better clinical management and informed decision making.

### Interventions

CHUM’s senior administrators first instructed the center for optimizing network flows (called COFR in French) to centralize and coordinate requests for complementary expertise and patient transfers to the burn unit. COFR operates like a call management center. It consists of a dozen nurse clinicians who systematize access to the hospital, support patient management, and forward requests for complementary expertise. COFR supports the needs of several departments, including critical care, vascular surgery, and addiction medicine. Then COFR was charged with governing physician discussions using a telemedicine mobile application. The Reacts^TM^ mobile application (Remote Education, Augmented Communication, Training and Supervision, by Innovative Imaging Technologies Inc., Montreal, Canada) was selected for this purpose. This app, which has been certified by the Quebec’s health authorities for remote collaborative communications, is HIPAA compliant and incorporates innovative tools like augmented reality for remote virtual guidance, supervision, and training. It is deployed in over 80 countries to support remote wound care, tele-ultrasound, teleconsultations with patients, interactive tele-surgical assistance, and remote procedural supervision, among others. In the present telemedicine project, Reacts^TM^ is to be used for secure file transfers, instant messaging, and video conference calls.

### Trial design and feasibility outcomes

As shown in Table 1, we developed a three-stage structured trial protocol which uses a mixed methods research design. To ensure completeness and quality of reporting, we referred to the SPIRIT (Standard Protocol Items: Recommendations for Interventional Trials) guideline [17] as well as Thabane and Lancaster’s [18] guidance on how to report protocols of pilot and feasibility trials.

**Table 1:** Trial phases, feasibility outcomes, and data sources

Phase	Feasibility outcomes	Data sources
Technical feasibility (participation of 1 referring hospital)	Technical glitches, bugs, or problems	Observation of simulated teleconsultation sessions
		Interviews with referring physicians at one hospital and medical specialists at the burn center
Organizational and human feasibility (participation of 4 referring hospitals)	Organizational and human challenges (e.g., alignment of telemedicine with work practices; acceptability of telemedicine to physicians)	Transcripts of all discussions during the teleconsultation sessions
		Follow-up interviews with referring physicians and medical specialists
Preliminary assessment of potential value (participation of 10 referring hospitals)	Perceived benefits (clinical outcomes, quality of discussions, and knowledge transfer)	Structured interviews with referring physicians and medical specialists
	Clinical outcomes: time to transfer; ventilation procedures; patient complications; mortality; length of ICU stay; and additional surgical procedures.	SIRTQ database and EHR systems
	Economic viability	Burn center’s budget. Quebec health insurance plan (RAMQ)

#### Phase 1: technical feasibility

The first phase will consist of evaluating the project’s technical feasibility in terms of various operational issues, such as access to a wireless communications network (a mobile or Wi-Fi network) in the emergency room, the user-friendliness of the telemedicine app, and the fluidity of discussions between the referring physician, the medical specialist, and COFR staff. The participation of a referring hospital with a high volume of patient transfers will be required. Technological feasibility will be evaluated through an iterative process in which the new work process and tools will be tested and adjusted accordingly. Various technical tests (login, connectivity, audio/video problems, etc.) will be performed through observations of all communications. Following these tests, physicians and COFR staff will be interviewed to assess the new work process and methods as well as the telemedicine mobile app’s strengths and weaknesses.

#### Phase 2: organizational and human feasibility

Once the technical issues are sorted out, the second phase will consist of evaluating the organizational and human feasibility of the project. To this end, the new workflow methods and tools will be deployed in four referring hospitals. During a 9-month period, referring physicians and medical specialists will discuss cases over the telemedicine platform. This material will enrich the discussions and reflections during the follow-up interviews to be conducted with all the participating physicians. The analysis grid used in this phase and phase 1 will be based on the World Health Organization’s telemedicine implementation model [19]. Based on this analysis, a committee will design and implement a change management strategy to deal with the organizational and human aspects of technological change. The goal is to prepare medical teams and help guide them through the transition until the new work practices and methods become routine [20, 21].

#### Phase 3: initial assessment of clinical and economic value

The third phase will consist of evaluating, over a period of 12 months, the clinical and economic value of the telemedicine platform that will be deployed to a subset of 10 regional hospitals (out of 73 referring sites) in early 2021. As in the previous phase, after each telemedicine session, a follow-up interview will be conducted with the pair of physicians involved. Participants will be asked to comment on the quality of their discussions as well as the perceived effects of telemedicine on the quality of case-based discussions and clinical decisions as well as on knowledge transfer (i.e., access to burn education). Next, several variables will be extracted from the SIRTQ database: (a) the rate of unjustified transfers, (b) the rate of unjustified ventilations, and (c) estimation differences. The quantities of unjustified procedures (i.e., transfers and assisted ventilations) will be analyzed as annual proportions for all the referring hospitals involved. Other outcomes available in the burn center’s or the referring hospitals’ EHR systems will be collected and analyzed, including patient complications, mortality, length of ICU stay, additional surgical procedures, and time to transfer to the burn center. Given that telemedicine will be gradually deployed to the 10 participating referring sites in 2021, data for the years 2019 and 2020 will serve as the baseline (pre-deployment) and will be compared with data for the years 2022 and 2023 (post-deployment). It is worth noting that the other referral burn center in Quebec, which is part of the Laval University Health Center, and 10 of its referring hospitals will serve as a control group. Telemedicine is not available at this referral center, and there is no plan to deploy it prior to the end of the present study. Hence, it will be possible to use the same data periods in both medical units. Last, a preliminary assessment of the economic viability of telemedicine will also be conducted using a cost-minimization approach [22].

### Sample size

In stage 1, the participation of 4 to 6 medical specialists and 6 to 10 referring physicians is expected. In the second stage, the participation of 8 to 10 pairs of physicians (referring physician and medical specialist) is anticipated. In stage 3, we expect to seek the participation of 10 to 15 pairs of physicians to assess the impact of telemedicine on the quality of case-based discussions and clinical decisions as well as on knowledge transfer.

### Data analysis

In the first two phases, qualitative data will be analyzed by applying deductive reasoning [23] to identify the technical, organizational, and human feasibility issues encountered. All interview materials will be audio-taped, stored, and transcribed verbatim. The analysis will be based on the World Health Organization’s telemedicine implementation model [19] which accounts for the technical, organizational, human, and economic barriers associated with telemedicine initiatives. In phase 3, the interview data will be analyzed through a process of inductive reasoning [23]. Next, quantitative indicators (e.g., rate of unjustified transfers, rate of unjustified ventilations) will be analyzed using descriptive statistics (mean, standard deviation). Last, the economic viability of telemedicine will be evaluated using a cost minimization approach [22]. The analysis will consider the difference between costs incurred and costs avoided [24]. Costs incurred will be the resources necessary to run the telemedicine operations, including the purchase of licences, the cost of training users, and the cost of operating COFR. The avoided costs will be determined conditional to a statistically significant reduction in the number of unjustified procedures in the post-deployment period. The average costs of patient transfers and assisted ventilations will be estimated with assistance from CHUM’s budget and economic performance unit. These estimates will include, but will not be limited to, the costs of travel, the stay at the burn center, administrative support, and support services. Consultations with medical specialists at the burn unit will also be used to estimate the cost of medical procedures, using the physicians’ invoicing manual published by the province’s health insurance authority as a reference.

## Discussion

Considering that trauma patients (including severe burn victims) have better outcomes when they receive timely treatment at a specialized center [25], that delays to definitive treatment have a negative impact on survival and long-term morbidity following a traumatic event [25], and that triaging burn patients represents a challenging task because severe burn injuries are rare [26], it becomes vital to connect providers at referring hospitals with medical experts for real-time advice on the early management of these patients. Telemedicine represents a solution to these challenges. Numerous studies have investigated the use of telemedicine in a variety of environments and patient populations, yet solid evidence on telemedicine usage in the initial management of severe burn victims is still lacking [27–30]. A recent rapid review of the literature on telemedicine in rural trauma care [31] reveals that telemedicine may improve patient diagnosis, streamline the process of transferring patients, and reduce length of stay, and that it has minimal impact on mortality and patient complications. Mixed findings were observed in terms of patient transfer times [31]. Another recent study showed that telemedicine increased efficiency of resource utilization, timely resuscitation, appropriate transfer of patients requiring admission, and real-time education [26].

The present pilot study aims to deepen our understanding of the barriers to and outcomes of telemedicine in the initial management of severe burn victims. We have reported a three-stage, structured approach to evaluating the feasibility, acceptability, and potential value of a telemedicine initiative in providing optimal care to severe burn patients in Quebec, Canada. We posit that when properly planned and implemented, telemedicine has the potential to significantly improve the clinical management and transfer of burn victims. To increase the chances of implementation success, phases 1 and 2 of the pilot study protocol are designed to identify the various technical, organizational, and human issues associated with the deployment of large telemedicine initiatives in this environment. Our feasibility analysis will guide or orient hospital administrators in charge of designing an effective change management strategy. The third phase of the protocol will provide a preliminary assessment of the clinical and economic value of the telemedicine initiative. We propose a mixed methods research approach for this assessment. As mentioned above, prior empirical findings related to the effects of telemedicine in the context of burn care are still scarce and mostly inconclusive (e.g., [32–36]). The pre-test/post-test control group design presented above shall provide solid empirical evidence on the nature and scope of the effects of telemedicine in this context.

The proposed study protocol has some limitations. First, it may not be possible to detect all the specific effects of COFR and the telemedicine app, as these interventions will be deployed concurrently. In response to the problems found during initial management, it has been decided to use a stay of less than 24 h as the threshold for determining an unjustified, unnecessary transfer of a burn patient. However, we are aware that the length of stay of a burn patient is often estimated based on a percentage of the total area affected [37, 38]. Measuring length of stay according to burn surface area percentage may not only be relevant in the context of per-operative follow-up, but also during the rehabilitation phase. This highlights another limitation of the proposed protocol: a focus on the early clinical management of burn victims. Future research could examine the impacts of telemedicine on other stages of the care continuum, such as the rehabilitation stage [39].

In conclusion, the telemedicine initiative described here is expected to yield positive and significant results that are relevant to a wide range of medical centers that are already using or are considering deploying similar technologies. The dissemination of study results will also be relevant locally, knowing that the CHUM’s administration intends to deploy telemedicine in other types of inter-facility transfers (e.g., for limb amputations, triage in response to a major disaster). The contribution of this pilot study lies in its ability to reveal technological, human, and organizational challenges and provide a preliminary assessment of the clinical and economic value associated with telemedicine in the specific context of acute care for severe burn victims.

## Supplementary information

**Additional file 1.** Details of the transfers to the Burn Victim Unit (year 2017). This file presents the distance from the Burn Victim Unit (km) and the number of patient transfers for each of the institutions included in the areas of Western Quebec in 2017.

## Data Availability

The datasets that will be generated during the study will be available from the corresponding author on reasonable request.

## Abbreviations

CHUM: University of Montreal Health Center (called CHUM in French)
SIRTQ: Système d’information du registre des traumatismes du Québec
COFR: Center d’optimisation des flux réseau
HIPAA: Health Insurance Portability and Accountability Act
RAMQ: Régie de l’assurance maladie du Québec
