# Supplementary material for: Early clinical management of severe burn patients using telemedicine: a pilot study protocol
Source: Pilot Feasibility Stud. 2020 Jul 4;6:93. doi: 10.1186/s40814-020-00637-7 (PMC7334850; doi:10.1186/s40814-020-00637-7)
Supplement: Supplementary file 1 — Additional file 1. Details of the transfers to the Burn Victim Unit (year 2017). This file presents the distance from the Burn Victim Unit (km) and the number of patient transfers for each of the institutions included in the areas of Western Quebec in 2017. [file 40814_2020_637_MOESM1_ESM.docx]

**Additional file 1.** **Details of the transfers to the Burn Victim Unit (year 2017)**

| **Areas of Western Quebec** | **Institutions** | | | **Distance from the Burn Victim Unit (km)** | **Number of patient transferts** |
| --- | --- | --- | --- | --- | --- |
| Abitibi – Témiscamingue | Hôtel-Dieu d'Amos Hospital | | | 596 | 2 |
|  | La Sarre Hospital | | | 689 | 0 |
|  | Rouyn-Noranda Hospital | | | 634 | 0 |
|  | Val-d'Or Hospital | | | 526 | 0 |
|  | Ville-Marie Hospital | | | 669 | 0 |
|  | Témiscaming-Kipawa Hospital | | | 582 | 0 |
| Estrie | Magog Hospital | | | 127 | 1 |
|  | Fleurimont Hospital | | | 158 | 0 |
|  | Lac-Mégantic Hospital | | | 246 | 0 |
|  | Asbestos Hospital | | | 169 | 1 |
|  | Hôtel-Dieu de Sherbrooke Hospital | | | 159 | 2 |
|  | Coaticook Hospital | | | 165 | 0 |
|  | Argyll Hospital | | | 153 | 0 |
|  | Youville Hospital | | | 158 | 0 |
| Lanaudière | Lanaudière Regional Health Centre | | | 102 | 6 |
|  | Pierre-Le Gardeur Hospital | | | 30 | 5 |
| Laurentians | Saint-Eustache Hospital | | | 52 | 5 |
|  | D’Argenteuil Hospital | | | 92 | 0 |
|  | Mont-Laurier Hospital | | | 239 | 2 |
|  | Laurentien Hospital | | | 105 | 6 |
|  | Rivière-Rouge Hospital | | | 180 | 3 |
|  | Saint-Jérôme Regional Hospital | | | 59 | 2 |
| Laval | Cité-de-la-Santé Hospital | | | 19 | 1 |
|  | Jewish Rehabilitation Hospital | | | 19 | 0 |
| Montérégie | Charles LeMoyne Hopital | | | 10 | 11 |
|  | Granby Hospital | | | 85 | 3 |
|  | Anna-Laberge Hospital | | | 48 | 0 |
|  | Haut-Richelieu Hospital | | | 43 | 3 |
|  | Brome-Missisquoi-Perkins Hospital | | | 95 | 2 |
|  | Honoré-Mercier Hospital | | | 60 | 6 |
|  | Suroît Hospital | | | 81 | 0 |
|  | Hôtel-Dieu de Sorel Hospital | | | 80 | 2 |
|  | Hôpital Pierre-Boucher Hospital | | | 14 | 4 |
|  | The Barrie Memorial Hospital | | | 88 | 1 |
| Montreal | Montreal Sacré-Cœur Hospital | | | 19 | 0 |
|  | Montreal General Hospital | | | 5 | 1 |
|  | Jean-Talon Hospital | | | 7 | 0 |
|  | Santa Cabrini Hospital | | | 11 | 0 |
|  | Centre Hospitalier Universitaire Sainte-Justine | | | 7 | 1 |
|  | Montreal Children's Hospital | | | 7 | 0 |
|  | Lakeshore General Hospital | | | 29 | 2 |
|  | Lachine Hospital | | | 16 | 0 |
|  | Rivières-des-Prairies Hospital | | | 18 | 0 |
|  | Donald Berman Maimonides Geriatric Centre | | | 13 | 0 |
|  | Institut Philippe-Pinel de Montréal | | | 22 | 0 |
|  | Montreal Neurological Hospital | | | 3 | 0 |
|  | Fleury Hospital | | | 12 | 0 |
|  | Institut de réadaptation Gingras-Lindsay | | | 8 | 0 |
|  | Montreal Mont Sinai Hospital | | | 12 | 0 |
|  | Verdun Hospital | | | 7 | 0 |
|  | Maisonneuve-Rosemont Hospital | | | 9 | 0 |
|  | Shriners Hospitals for Children | | | 7 | 0 |
|  | Montreal Chinese Hospital | | | 1 | 0 |
|  | Richardson Hospital | | | 11 | 0 |
|  | Lasalle Hospital | | | 16 | 0 |
|  | St. Mary's Hospital Centre | | | 8 | 2 |
|  | The Douglas Institute | | | 10 | 0 |
|  | Villa Medica Rehabilitation Hospital | | | 1 | 0 |
|  | McGill University Health Centre | | | 7 | 0 |
|  | Institut universitaire en santé mental de Montréal | | | 10 | 0 |
|  | Marie-Clarac Hospital | | | 14 | 0 |
|  | Jewish General Hospital | | | 8 | 6 |
|  | Sainte-Anne Hospital | | | 42 | 0 |
|  | Notre-Dame Hospital | | | 0 | 3 |
|  | Royal Victoria Hospital | | | 7 | 2 |
| Northern Quebec | Chibougamau Health Centre | | | 694 | 0 |
|  | Isle-Dieu Health Centre | | | 730 | 0 |
|  | Lebel Health Centre | | | 620 | 0 |
|  | Radisson Health Centre | | | Air | 0 |
|  | René-Ricard Health Centre | | | 712 | 0 |
|  | Inuulitsivik Health Centre | | | Air | 1 |
|  | Ungava Tulattavik Health Centre | | | Air | 1 |
|  | Chisasibi Regional Hospital Centre | | | Air | 0 |
|  | Eastmain CMC | | | Air | 0 |
|  | Mistissini CMC | | | Air | 0 |
|  | Nemaska CMC | | | Air | 0 |
|  | Oujé-Bougoumou CMC | | | Air | 0 |
|  | Waskaganish CMC | | | Air | 0 |
|  | Waswanipi CMC | | | Air | 0 |
|  | Wemindji CMC | | | Air | 0 |
|  | Whapmagoostui CMC | | | Air | 1 |
| Outaouais | Hull Hospital | | | 205 | 1 |
|  | Pontiac Hospital | | | 277 | 0 |
|  | Maniwaki Hospital | | | 301 | 1 |
|  | Gatineau Hospital | | | 199 | 0 |
|  | Wakefield Memorial Hospital | | | 236 | 0 |
|  | Geriatric day care Hospital | | | 198 | 0 |
|  | Pierre Janet Hospital Centre | | | 205 | 0 |
|  | Papineau Hospital | | | 170 | 3 |
|  | | Transfert from areas outside of Western Quebec | | | 2 |
|  | | Transfert from outside of Quebec | | | 5 |
|  | | Direct transportation from trauma site | | | 43 |
|  | | Total 2017 | | | 143 |
| Community Miyupimaatisiiun center (CMC)  Aeromedical evacuation (Air) | | |  | | |
